# Association of SO_2_/CO exposure and greenness with high blood pressure in children and adolescents: A longitudinal study in China

**DOI:** 10.3389/fpubh.2023.1097510

**Published:** 2023-05-25

**Authors:** Yi Zhang, Shuo Chen, Li Chen, Yu Wu, Jing Wei, Tao Ma, Manman Chen, Qi Ma, Jieyu Liu, Xinxin Wang, Yi Xing, Lijuan Wu, Weiming Li, Xiangtong Liu, Xiuhua Guo, Jun Ma, Yanhui Dong, Jingbo Zhang

**Affiliations:** ^1^Institute of Child and Adolescent Health, School of Public Health, Peking University, National Health Commission Key Laboratory of Reproductive Health, Beijing, China; ^2^Beijing Physical Examination Center, Beijing, China; ^3^School of Population Medicine and Public Health, Chinese Academy of Medical Sciences/Peking Union Medical College, Beijing, China; ^4^Department of Atmospheric and Oceanic Science, Earth System Science Interdisciplinary Center, University of Maryland, College Park, MD, United States; ^5^School of Public Health and Management, Ningxia Medical University, Yinchuan, China; ^6^Department of Epidemiology and Health Statistics, Capital Medical University School of Public Health, Beijing, China; ^7^Beijing Municipal Key Laboratory of Clinical Epidemiology, Capital Medical University, Beijing, China

**Keywords:** sulfur dioxide, carbon monoxide, blood pressure, greenness, body mass index

## Abstract

**Introduction:**

We aimed to investigate the association between greenness around schools, long-term gaseous air pollution exposure (SO_2_ and CO), and blood pressure in children and adolescents.

**Methods:**

From 2006 to 2018, a total of 219,956 Chinese children and adolescents aged 7–17 years in Beijing and Zhongshan were included in this longitudinal study. Annual average concentrations of SO_2_ and CO and the mean values of normalized difference vegetation index around schools were calculated. We used the generalized estimation equation model, restricted cubic spline model, and Cox model to analyze the health effects.

**Results:**

Among all the subjects, 52,515 had the first onset of HBP. During the follow-up, HBP's cumulative incidence and incidence density were 23.88% and 7.72 per 100 person-year respectively. Exposures to SO_2_ and CO were significantly associated with SBP [β = 1.30, 95% CI: (1.26, 1.34) and 0.78 (0.75, 0.81)], DBP [β = 0.81 (0.79, 0.84) and 0.46 (0.44, 0.48)] and HBP [HR = 1.58 (1.57, 1.60) and 1.42 (1.41, 1.43)]. The risks of HBP attributed to SO_2_ and CO pollution would be higher in school-aged children in the low greenness group: the attributable fractions (AFs) were 26.31% and 20.04%, but only 13.90% and 17.81% in the higher greenness group. The AFs were also higher for normal-BMI children and adolescents in the low greenness group (AFs = 30.90% and 22.64%, but 14.41% and 18.65% in the high greenness group), while the AFs were not as high as expected for obese children in the low greenness group (AFs = 10.64% and 8.61%), nor was it significantly lower in the high greenness group (AFs = 9.60% and 10.72%).

**Discussion:**

Greenness could alleviate the damage effects of SO_2_/CO exposure on the risks of HBP among children and adolescents, and the benefit is BMI sensitivity. It might offer insights for policymakers in making effective official interventions to prevent and control the prevalence of childhood HBP and the future disease burden caused by air pollution.

## 1. Introduction

High blood pressure (HBP) in children was once considered a rare disease, but it is now a public health concern worldwide (1). Today, childhood HBP affected over 1.13 billion people worldwide as it significantly increases the risks of heart attack, stroke, and other complications (2). In recent years, the incidence of HBP has increased constantly in developing countries. Environmental and lifestyle changes might contribute to its prevalence (3, 4).

Evidence indicated that air pollutants sulfur dioxide (SO_2_) and carbon monoxide (CO) might be important risk factors for hypertension (5, 6) and other CVDs in adults (7, 8). Animal experiments also showed that short- and long-term exposure to SO_2_ could cause functional damage to the cardiovascular system (9, 10). Its possible mechanisms could be explained by oxidative stress, alterations in the autonomic nervous system, or ion concentration change in body fluids (9, 11, 12). However, this evidence was limited (13) compared to similar studies about PM_10_, PM_2.5_, ozone, and NO_2_, especially in cohort studies and large populations of children and adolescents in developing countries (14–19). Moreover, the limited evidence supported inconsistent conclusions. For example, 5-day short-term exposure to SO_2_ was reported to have no association with BP increase (20). The long-term exposure investigation, however, observed a strong correlation between SO_2_/CO exposure and hypertension (16). Greenness plays another important role in cardiovascular health, according to the assessment of the beneficial impact of greenspace on a dozen health outcomes, including BP (21). Cross-sectional and retrospective studies mostly focused on the potential benefits of plants to attenuate the respiratory and cardiovascular health risks of PM_10_, PM_2.5_, and NO_2_ pollution in adults or seniors (22–26). Few of them focused on SO_2_ and CO. A cross-sectional study in Northern China found a 10-μg/m^3^ increase in SO_2_ was responsible for a 2.43% increase in mean arterial pressure among adults. These identified harmful effects of SO_2_ mainly occurred among people who lived in low-greenness environments (27). Subgroup analysis of greenness may provide a concise illustration of the role of greenness on health outcomes. Although the association may be non-linear, higher greenness is probably to have a more positive effect on hypertension (28). In terms of biological mechanisms, exposure to better green space can increase immunoregulation, lowering the risk of inflammatory and cardiovascular diseases. Greenness can also improve physical activities and reduce noise effects (29) to counteract the negative effects of environmental pollution by improving stress resilience (30). In addition, children and adolescents of different genders, ages and BMIs have various physiological, psychological, and socio-environmental differences. The blood pressure in different subgroups may be influenced by growth and development characteristics, for example, sex-related differences in hormone secretion, age-related differences in puberty timing (31), obesity-related differences in endocrine regulation/insulin resistance, and environment sensitivity (32, 33). Previous findings on these subgroups' sensitivity to SO_2_, CO and greenness exposure are limited (18, 27) and therefore further validation and supplementation are still needed.

In this multicenter, longitudinal, prospective open cohort study in China, we hypothesized that SO_2_ and CO were risk factors for HBP in school-aged children and adolescents, while greenness protected blood pressure. We also aimed to investigate whether a high level of greenness can reduce this risk, and whether different BMIs groups had different sensitivities.

## 2. Materials and methods

### 2.1. Design and population

This study was based on an open cohort covering all school-age students attending primary, junior, and senior high schools aged 7–17 years in Beijing and Zhongshan from 2006 to 2018. They were enrolled through an annual medical examination survey, similar to a census for local children and adolescents, except for school dropouts, as described in detail in the previous study (34). Students were included in the study from 1,839 schools, and we matched individual data for each year based on unique codes. In this open cohort, participants were free to enter and leave as they liked. There was no specific selection of participants and no strict definitions of inclusion and exclusion criteria. Participants were included in the open cohort if they had their first medical examination records from their first year of primary school to their second year of high school.

A total of 3,290,046 participants entered the open cohort. During data processing, we excluded participants with missing information on weight, height, birthday, and blood pressure (*N* = 261,122), those with abnormal data (*N* = 920,095), and those diagnosed with HBP at baseline or with only one record of blood pressure (*N* = 1,484,725). Therefore, the eligible participants included in the study had more than twice completed annual medical examinations. The annual follow-up with medical check-ups was usually between September–November or April–June. We further excluded another 404,148 subjects with missing data on SO_2_/CO/NDVI around schools after linking the annual physical examination survey to the gaseous pollution data. Eventually, we enrolled 219,956 participants in the final analysis (Beijing: 46,652, Zhongshan: 173,304). The subjects were followed up annually until the onset of HBP, loss, and the end of the study, whichever came first. Children and adolescents are automatically out of the cohort when they reach the age of 18 or graduate from high school. The flow chart is shown in Supplementary Figure A1 and the characteristics of the included and excluded participants are shown in Supplementary Table A1. The study was approved by the Biomedical Ethics Committee of Peking University Health Science Center (Reference Number: IRB00001052-20033).

### 2.2. Anthropometric measurement and outcomes definition

Anthropometric data, including height (cm), weight (kg), and systolic and diastolic blood pressure (SBP, DBP; mmHg) were measured and recorded by trained physicians. Height was accurately measured to 0.1 cm with portable stadiometers and weight was accurately measured to 0.1 kg with a standardized scale. All participants were required to stand barefoot with light clothing, naturally straight torsos, straight heads, and eyes straight in front, as well as upper limbs hanging naturally and the legs straight. Height and weight were measured twice, and the mean was recorded. Body Mass Index (BMI, kg/m^2^) is calculated as the weight (kg) divided by the square of height (m). BMI groups were categorized into three levels (normal weight, overweight, and obesity) according to growth reference data for 5–19 years old children from the WHO definition (35).

Right arm brachial BP in a sitting position was used for BP measurement, with appropriate cuff sizes according to the actual situation. Auscultation mercury sphygmomanometer was uniformly used. SBP (mmHg) and DBP (mmHg) were measured 3 times, respectively, by Korotkoff I sound and V sound (vanishing sound), and the average value was calculated and recorded. After each measurement, the cuff was loosened for about 2 mins. Systolic and diastolic high blood pressure (SHBP and DHBP) were defined as SBP and DBP above or equal to the 95th percentile of the reference population by age, sex, and height. HBP was defined as the presence of either SHBP or DHBP (36).

### 2.3. Assessment of SO_2_/CO concentration and estimation of greenness

**Figure 1:**
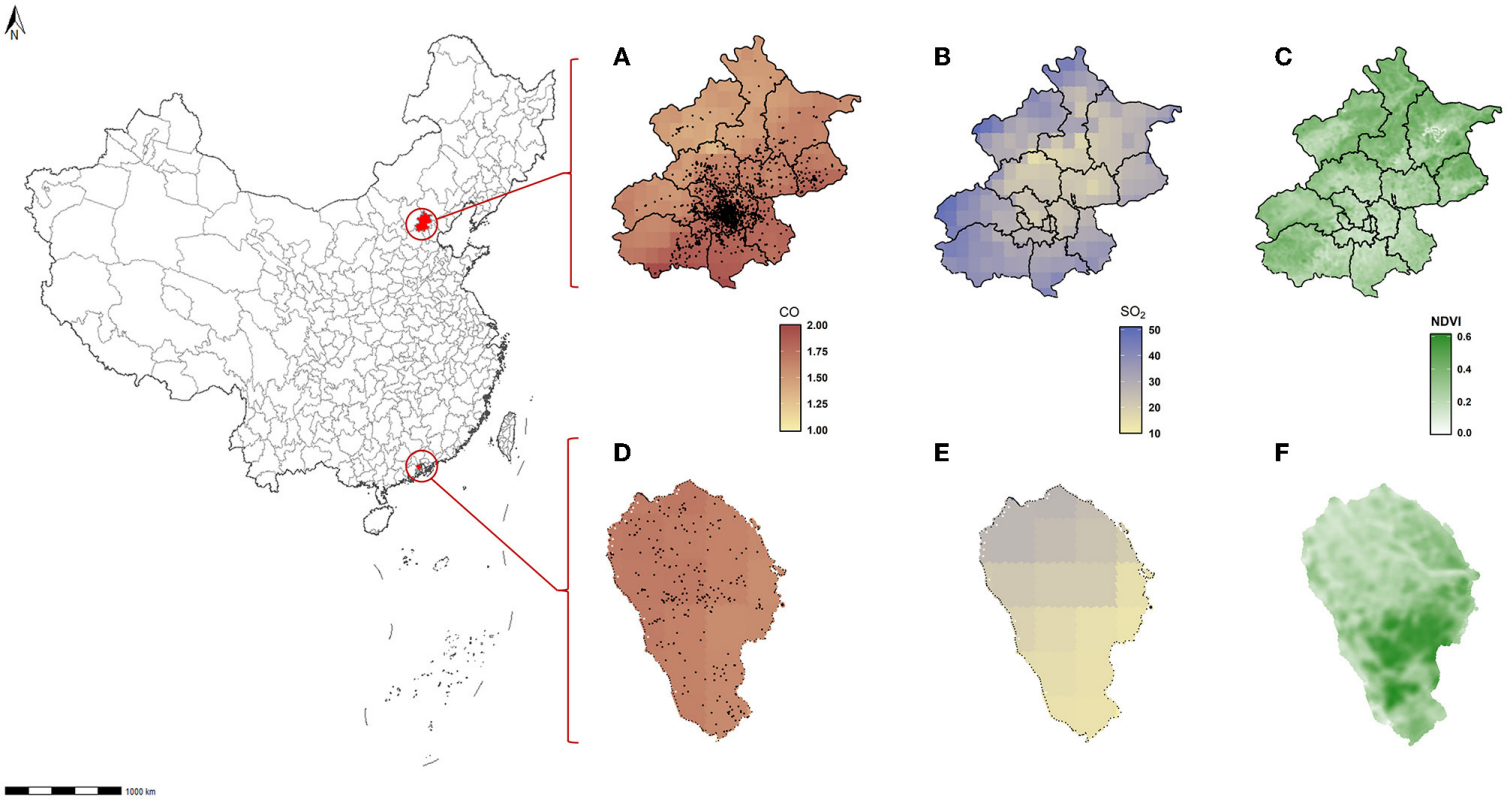
Location of schools surveyed, the SO_2_/CO concentration, and NDVI distribution in Beijing and Zhongshan. **(A)** Mean concentration of CO in Beijing 2014. **(B)** Mean concentration of SO_2_ in Beijing 2014. **(C)** Annual NDVI in Beijing 2014. **(D)** Mean concentration of CO in Zhongshan 2014. **(E)** Mean concentration of SO_2_ in Zhongshan 2014. **(F)** Annual NDVI in Zhongshan 2014.

SO_2_ and CO concentrations were calculated at a spatial resolution of 10 km from 2014 to 2018 in the CHAP dataset (available at: https://weijing-rs.github.io/product.html). The dataset is generated using a space-time extremely randomized trees (STET) model from big data, including satellite remote sensing, meteorology, multi-resolution emission inventory, and land utilization data (37, 38). The annual average concentrations of SO_2_ and CO around schools 1 year before the occurrence of HBP or the end of follow-up were used to indicate gaseous pollutants exposure. The distributions of SO_2_ and CO in Beijing and Zhongshan in 2014 are shown in Figure 1.

Greenness was estimated with a normalized difference vegetation index (NDVI) with a resolution of 30 m by 30 m. The formula of NDVI is: $NDVI=\frac{\text{Near Infrared }-\text{ RED}}{\text{Near Infrared }+\text{ RED}}$, where Near Infrared (NIR) denotes the land surface reflectance of near-infrared, and RED denotes the surface reflectance in red regions of the electromagnetic spectrum. NDVI values range between −1.0 to 1.0. It is assumed that a negative value indicates a water area, and a value close to 0.0 may be bare ground without vegetation (39). A low positive value indicates a barren area with vegetation, a medium positive value indicates low vegetation, and a high positive value indicates dense vegetation such as trees. The annual mean of NDVI within a 1 km radius of each school in 1 year before the onset of HBP or the end of follow-up was used as an individual's greenness exposure. A higher NDVI value indicates a higher green vegetation density. In our study, NDVI values ranged from 0.1023 to 0.7199, indicating various green environments surrounding schools. The data was divided into two NDVI levels using the median (0.2816) as a cut-off line.

### 2.4. Statistical analysis

Categorical variables were described by frequencies and rates, and continuous variables were described by means and standard deviation (SD).

The cumulative incidence of HBP was calculated as the number of new HBP during the follow-up period divided by the total number of participants. The incidence density (ID) was calculated by: ID = $\frac{N_{new}}{\sum \left(Y_{i}\times n_{i}\right)}$, where *N*_*new*_ was the number of new HBP incidents during the follow-up, *Y*_*i*_ was the year between entry into the cohort and the onset of HBP, loss, and the end of the study, whichever came first; *n*_*i*_ represented the number of participants corresponding to *Y*_*i*_.

**Figure 2:**
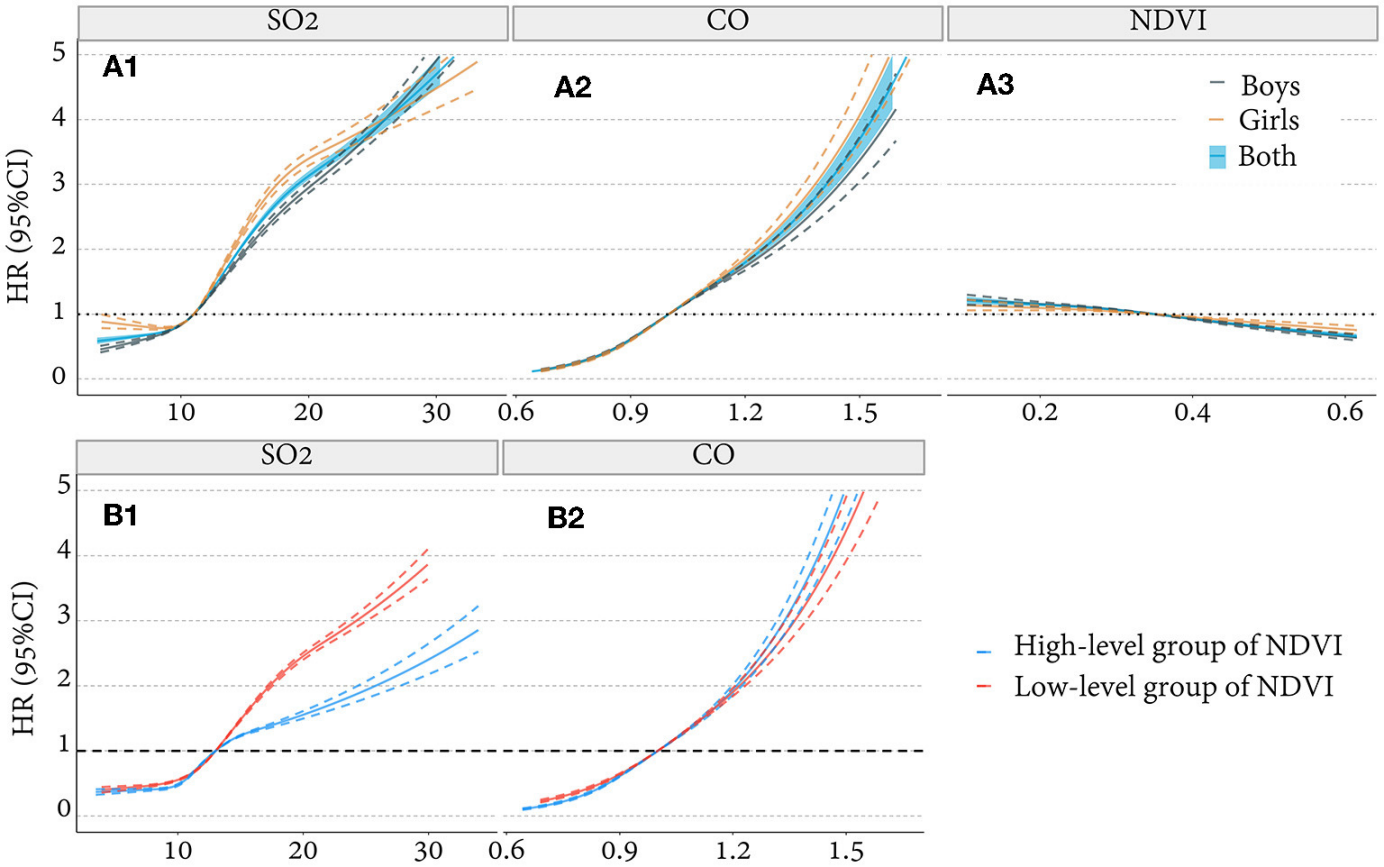
The non-linear correlation between SO_2_/CO/NDVI and HBP (binary outcome variable). **(A1, A2)** Association of SO_2_/CO with HBP adjusted for age, BMI, greenness, and city and stratified by sex. **(A3)** Association between NDVI and HBP adjusted for age, BMI, pollution, and city and stratified by sex. **(B1, B2)** Association of SO_2_/CO with HBP adjusted for age, sex, BMI, and city and stratified by greenness level. HR, hazard ratio.

We created a Cox regression with restricted cubic splines (RCS, Figure 2) to describe the qualitative relationship between SO_2_/CO/greenness and the HBP risks. The Event Status of participants was recorded as “1” when they were diagnosed with HBP, and “0” when HBP subsided or was not present until the end of the study. Event Time was calculated in years, from the entry of the cohort to the latest event status record. The Cox model can also evaluate the hazard ratio (HR) of HBP attributable to SO_2_/CO/NDVI by sex. We also considered that a participant's blood pressure at annual follow-up was correlated with that at baseline. Although participants having HBP at baseline were excluded, it is still necessary to consider the impact of multiple measurements on risk assessment, which potentially overestimated the role of risk factors on outcomes. We, therefore, used the generalized estimation equation (GEE) model to address the problem of correlation between individuals repeating multiple measurements at baseline, follow-up, and ending. For estimating the quantitative association between SO_2_/CO/NDVI and SBP/DBP, β represents the regression coefficient obtained after adjusting age, sex, BMI, and city, results of which could also be validated with Cox regression results for HBP. We also tested for interactions using GEE regression with robust standard errors. Given interactions between NDVI and SO_2_/CO (Supplementary Table A2), we further divided greenness levels into low and high NDVI groups based on the median value of NDVI and created a Cox model to evaluate the HR of HBP induced by NDVI grouped pollutants. The attributable fraction (AF) of SO_2_ or CO-induced HBP risk and the whole samples from the dynamic cohort were calculated to estimate the benefits of SO_2_ and CO control in reducing HBP at different greenness levels. The calculation methods were shown in previous research, $AF=1-\frac{1-\text{S}0\text{t}}{1-\text{St}}$, where S_0t_ denoted the counterfactual survival function for the event if the exposure was eliminated at baseline and S_t_ denoted the factual survival function (34). Covariates in our models included age, sex, BMI, and city. Additionally, we considered that the individuals with different BMIs have different sensitivity to the effect of environmental exposure on blood pressure and that this difference is important, so we performed a subgroup analysis for BMI and obtained as a secondary outcome.

All statistical analyses were completed using R version 4.1.1. All *p*-values were two-tailed, and < 0.05 was considered statistically significant unless otherwise stated.

## 3. Results

There were 219,956 children and adolescents without HBP at the baseline included in the analysis, 46,652 in Beijing and 173,304 in Zhongshan. The characteristic of the study subjects is displayed in Table 1. The mean age was 12.9 (SD: 2.89), and the mean NDVI was 0.30 (SD: 0.08). The average time of follow-up was 3.09 years. Mean SO_2_ and CO concentrations were 12.27 (SD: 3.93) μg/m^3^ and 0.97 (SD: 0.14) mg/m^3^. Among the participants without HBP at baseline, 52,515 were identified with new HBP during the follow-up, the cumulative incidence was 23.88%, and an incidence density was 7.72 per 100 person-year. The levels of NDVI exposure, BMI, and SO_2_ and CO concentrations were higher, but the average age, height, and weight were lower in the HBP group than in the non-HBP group (*p* < 0.001). The location of survey schools in Beijing and Zhongshan, their SO_2_ and CO concentration distribution and the NDVI levels in 2014 are shown in Figure 1.

**Table 1:** Characteristics of children and adolescents across the follow-up analysis.

**Characteristics**	**All^†^**	**Non-HBP^†^**	**HBP^†^**	***P* value**
	***n* = 219,956**	***n* = 167,441**	***n* = 52,515**	
Sample, *n* (%)				
Girls	96,225 (43.75)	73,426 (43.85)	22,799 (43.41)	0.079^#^
Boys	123,731 (56.25)	94,015 (56.15)	29,716 (56.59)	
Age (years); mean (SD)	12.9 (2.89)	13.19 (2.90)	11.96 (2.66)	< 0.001^*^
Years of follow-up; mean (SD)	3.09 (1.98)	3.25 (2.01)	2.57 (1.75)	< 0.001^*^
Height (cm); mean (SD)	153.54 (15.46)	154.93 (15.28)	149.11 (15.19)	< 0.001^*^
Weight (kg); mean (SD)	45.65 (15.68)	46.28 (15.39)	43.65 (16.43)	< 0.001^*^
BMI (kg/m^2^); mean (SD)	18.80 (3.82)	18.75 (3.69)	18.98 (4.19)	< 0.001^*^
NDVI^*^100; mean (SD)	30.05 (8.49)	30.10 (8.57)	29.90 (8.23)	< 0.001^*^
SO_2_ (μg/m^3^); mean (SD)	12.27 (3.93)	11.88 (3.66)	13.53 (4.48)	< 0.001^*^
CO (mg/m^3^); mean (SD)	0.97 (0.14)	0.97 (0.14)	0.99 (0.12)	< 0.001^*^

BMI, body mass index; NDVI, Normalized difference vegetation index. NDVI^*^100 (mean, SD), SO_2_ (mean, SD), and CO (mean, SD) represent the 1-year concentrations of NDVI, SO_2_, and CO (1 year before the endpoint of each participant), respectively. ^#^Chi-Square test; ^*^t-test.^†^There were 219,956 children and adolescents without HBP at the baseline included in the analysis, and 167,441 and 52,515 children and adolescents were identified with non-HBP and new HBP during the follow-up period.

We analyzed the non-linear correlation of SO_2_ and CO with the risk of HBP after adjusting age, sex, BMI, greenness, and city. We found that the risk of HBP in children and adolescents increases with the concentration of SO_2_ and CO (Figures 2A1, A2), Cox model with RCS. Meanwhile, the association of HBP risk with SO_2_ exposure was lower in high-greenery areas than in low-greenery areas, but this was not observed for CO exposure (Figures 2B1, B2), Cox model with RCS. We also explored a relationship between greenness and HBP, that risk of HBP decreases with the increase of NDVI (Figure 2A3).

**Figure 3:**
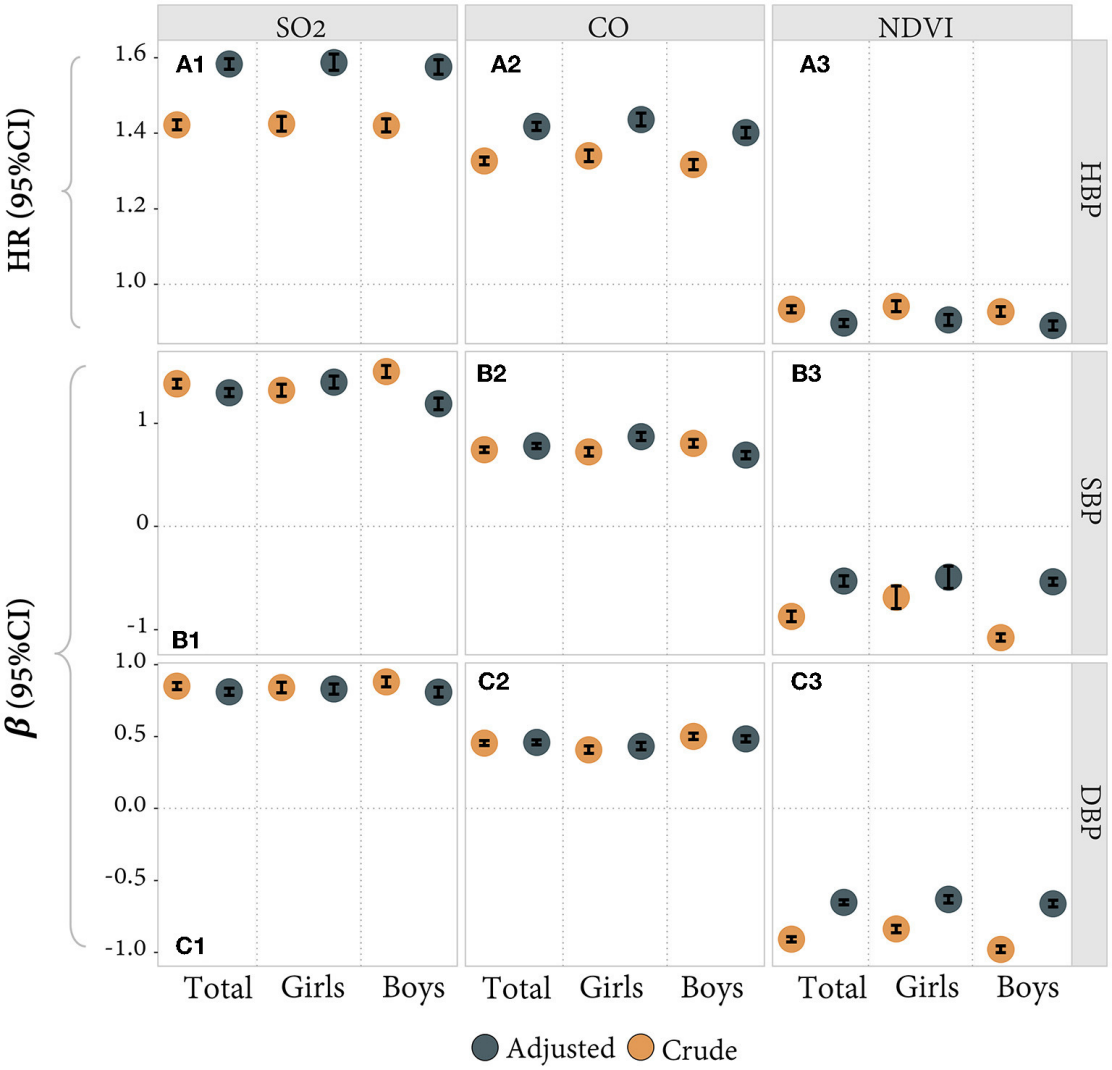
**(A1–A3)** The quantitative correlation between SO_2_/CO/NDVI and HBP (binary outcome variable and Cox model). **(B1–B3, C1–C3)** The quantitative association between SO_2_/CO/NDVI and SBP/DBP (continuous outcome variable and GEE model). Estimates adjusted for age, BMI, and city and stratified for sex (total estimates also adjusted for sex). β = estimated coefficient. HR, hazard ratio.

Based on the above preliminary results, we further analyzed the quantitative association of SO_2_, CO, and NDVI with BP levels and HBP risks, as illustrated in Figure 3. We found that the concentration of SO_2_ and CO positively correlated with SBP and DBP levels and HBP risks. It also had a higher effect on SBP: increased SO_2_ and CO exposure were significantly associated with SBP levels [β = 1.30 and 0.78, 95% CI: (1.26, 1.34) and (0.75, 0.81)] and were less associated with DBP levels [β = 0.81 and 0.46, 95% CI: (0.79, 0.84) and (0.44, 0.48)]. The HR of HBP was 1.58 and 1.42 respectively [95%CI: (1.57, 1.60) and (1.41, 1.43)]. NDVI was negatively correlated with SBP and DBP. Therefore, it could protect HBP: an increase in NDVI was significantly associated with SBP [β = −0.53, 95% CI: (−0.56, −0.49)] and DBP levels [β = −0.65, 95% CI: (−0.67, −0.63)], and the HR of HBP was 0.90 [95% CI: (0.89, 0.91)].

**Figure 4:**
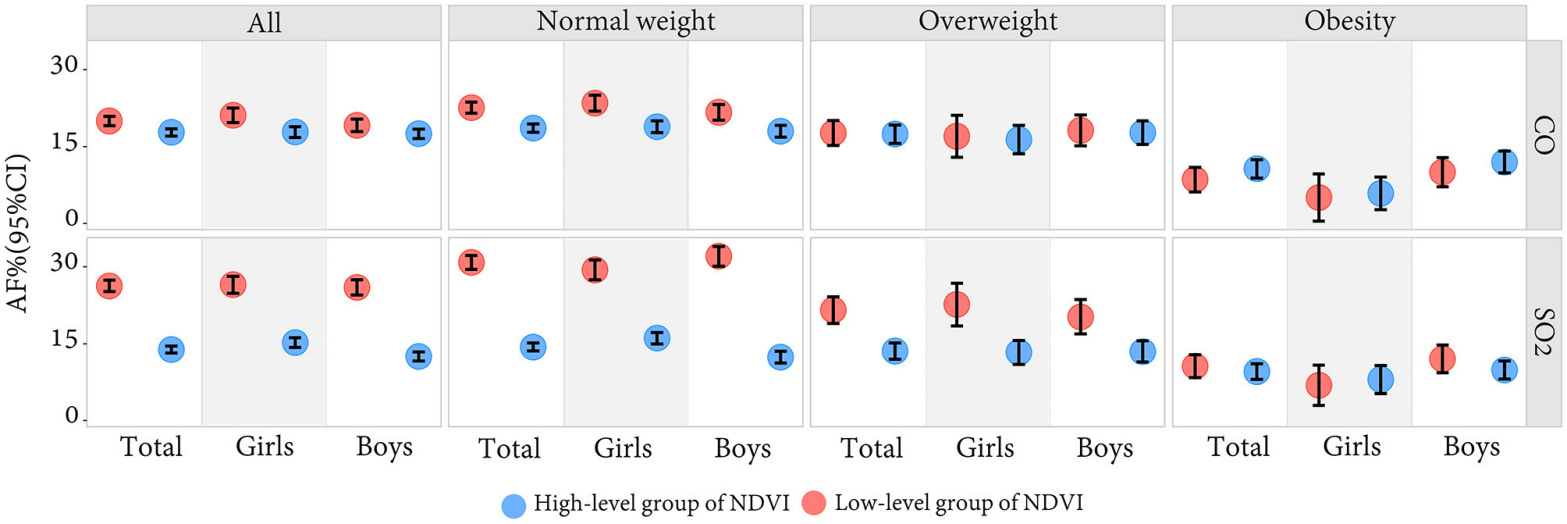
The attributable fraction of SO_2_ and CO on HBP in the low-level group and the high-level group of greenness by sex and BMI.

AF values were used to evaluate the theoretical benefits of HBP reduction through improving SO_2_ and CO among children and adolescents. The results showed that eliminating SO_2_ and CO exposure around schools could get a considerable theoretical benefit, but the benefit could vary by the levels of greenness. Improvement of SO_2_ and CO exposure benefits more in the low-level group of NDVI than in a high-level group of NDVI. The lower greenness group had a higher risk of HBP induced by SO_2_ and CO pollution. Figure 4 shows that AF values of HBP risks attributable to SO_2_ and CO in the low NDVI group of areas were 26.31% and 20.04% respectively, which were higher than those in a high NDVI group of areas (13.90% and 17.81%).

We also did a stratified analysis by BMI. Those who live in low-level green areas with normal nutritional status obtain great theoretical benefits. For obese participants, there was no significant difference between low and high-level greenness in the AFs of SO_2_ and CO. As shown in Figure 4, SO_2_ and CO-induced HBP risk was higher for normal-weight children and adolescents with a low-level greenness, the AFs of which were 30.90% and 22.64%, while only 14.41% and 18.65% for those living with a high-level greenness. However, the risk of HBP attributable to SO_2_ and CO was not as high as expected for obese children in a low-level greenness (AFs = 10.64% and 8.61%), nor was it significantly lower in the high-level greenness (AFs = 9.60% and 10.72%).

## 4. Discussion

Previous studies indicated that HBP in childhood or adolescence was a significant predictor for adult HBP and cardiovascular diseases (40, 41). However, the association of greenness and long-term SO_2_/CO exposure with childhood BP has yet to be studied in depth, particularly in a longitudinal study with potential causal inference. As far as we know, this was the first study that used a longitudinal, two-center, dynamic cohort in China to confirm that greenness can alleviate the damage effects of SO_2_/CO exposure on the risks of HBP among children and adolescents. The study also approved that improving individual exposure to SO_2_/CO can significantly benefit those with low-level greenness and normal weight. Our findings supported the reduction of the HBP risks among children and adolescents by improving greenness and reducing gaseous pollution around schools by a variety of measures.

Our findings were consistent with most previous relevant studies (6, 27, 39). For instance, a cross-sectional study of around 10,000 children from seven northeastern cities in China found that higher greenness levels around schools significantly lower the risk of childhood HBP (42). Another cross-sectional study found that high levels of SO_2_ and CO increased arterial blood pressure and HBP among children aged 5–17 in northeastern China (16). In addition, a two-decade population-based study in Tehran revealed that diastolic blood pressure was more sensitive to CO while SBP was more sensitive to SO_2._ They found that adults exposed to higher SO_2_ pollution had a significantly higher risk of HBP than those exposed to CO (14). Combined with our study, we suggested that children might be more sensitive to CO air pollution than adults. Another study on the interaction between obesity and air pollution on BP in Chinese children confirmed that children's obesity amplified the effects of long-term air pollution on BP (15). The result suggested that the BP effects of air pollution might be harder to eliminate in obese children than in normal-weight children. This further confirmed the plausibility of our findings. However, several other findings are inconsistent with ours. A nationwide cross-sectional investigation in China found that increases in greenness were associated with reductions in SBP and DBP (39), but not in our study. The reasons for that might be due to the sample distribution, and greenness measurement differences. Our study filled a gap in the pre-cardiovascular hazard caused by specific gaseous pollutants in cohort studies of 7–17 years-old children and adolescents compared to previous studies. We further found that improving air pollution according to greenness level is a specific healthy measure, and theoretical benefits vary with children of different body sizes.

Viewpoints vary on the mechanism underlying HBP or BP affected by gaseous pollutants. One theory posited that gaseous (NO_x_, SO_2_, CO, and O_3_) and particle (PM_10_ and PM_2.5_) pollution triggers pulmonary oxidative injury and systemic inflammation, leading to oxidative stress, with consequences of endothelial injury, vasoconstriction, thrombosis, and changes in blood pressure (43). Another study suggested that pollutants activate respiratory sensory nerves, affecting airway receptors, baroreceptors, and chemoreceptors, thereby modifying the autonomic nervous system control of BP (44). There were also several perspectives on the effect mechanisms of greenness on BP, such as reducing stress, improving physical activity, and reducing respiratory diseases (45–48). We hypothesized that greenness could also control BP by changing pollution levels. Through analyzing individual air quality components or different geographical regions in China, we discovered that greenness had a prominent role in improving urban air quality, especially in northern China. Its contribution can reach 16.2% (49). Studies demonstrated that trees could direct filter air pollutants, such as SO_2_, which are absorbed mainly through stomata. Absorption is not the only way in which plants improve air quality. The higher the tree canopy cover, the better the barrier effect, and the more pollutant mixtures from high in the air can be limited. Tree species, plants cover rate, length of the leaf season, pollution concentration, and precipitation in different cities will all affect the air purification of greenness, thus bringing about different levels of health benefits (49–53). However, for obese children, the function of greenness is very limited in eliminating the pollutants-related HBP risk attributed to pollutants. These findings and mechanisms indicated that targeted measures should control the individual pollutants in different areas and for children with different BMIs.

In addition to the above findings, secondary results are shown in the above figures and Supplementary Appendix. We found that greenness had a protective effect on participants' blood pressure. The mechanism follows: green plants absorb SO_2_ and CO primarily *via* leaf stomata and reduce air temperatures by transpiration, influencing microclimate, which can then promote physical activity and social engagement, and link to mental health benefits (50, 54, 55), thus bringing physical health benefits. Our study also investigated the differences in results between boys and girls, in different ages, as shown in Figures 2–4 and Supplementary Figure A2. There was an obvious difference between boys and girls on the nonlinear associations of SO_2_, CO, and greenness with the risk of HBP, indicating that girls are more subjected to pollutants and greenness differences than boys. The participants were grouped into four age groups every 3 years as an interval and analyzed in subgroups. The group of 7–9 years old was more sensitive than the other groups. The association between CO and HBP outcomes was weaker in the 13–15 years old group, their association of NDVI with HBP was even the opposite. The result might be because of the indoor and outdoor activity time among children with academic pressure. However, no detailed age sensitivity to gaseous pollutants has been reported in previous studies. In addition, two typical northern and southern cities were included in our study to reflect China's overall situation in and to improve the result scalability. Considering the differences between cities, we also tested the data for both cities separately. Although the two cities may have some differences in detail, the overall results appear to be consistent with the combined results after we adjusted for the city in our main results, as shown in Supplementary Figures A7, A8. The blood pressure hazards caused by SO_2_, and CO pollution were similar in Zhongshan. Still, the greenery protective effects were more stable across genders in Zhongshan than in Beijing, probably due to pollutant proportions and the plant species in the two cities. Therefore, we also did some sensitivity analysis, as shown in Supplementary Figures A3–A8.

This study has the following strengths: first, we used a dynamic longitudinal cohort covering almost all school-age students for 12 years. Second, this study focused on northern and southern cities in China, making the results more representative and balanced. Third, we used the restricted cubic spline analyses to explore the non-linear association between exposure and ending, which might be more relevant to reality. At last, in addition to exploring the independent association between gaseous pollutants and green space with BP separately. We also assessed the benefits of improving a single pollutant under different greenness conditions to provide theoretical ideas for policy development.

There were still some limitations: firstly and most importantly, we used a convenient sample to develop a cohort study and only collected physical examination and environmental data. No family background or lifestyle habits were included. As a result, our study did not investigate some confounders, like salt intake, parents' education level, temperature, and traffic noise, which might lead to fluctuation in the theoretical benefits of greenness (42, 56, 57). Secondly, we adopted annual mean air pollutants concentrations and NDVI at the school level. The inherent limitations of NDVI did not allow us to distinguish between plant types, which might also affect health differently. And the home addresses collected in our study were imprecise, but the school addresses were accurate to latitude and longitude coordinates. Although students may spend even time at school and home, the local climatic and geographic conditions were similar. As a result, we used the school environment as individual exposure. Thirdly, we assumed that students included in our study did not change schools. Individuals may still develop HBP after exposure, but we did not follow up on this situation, so we might underestimate the incidence of HBP in our study. In addition, a one-time BP value is not sufficient to confirm the HBP, three repeated visits on different occasions would be more accurate. Fourth, our study did not consider children who dropped out of school; therefore, it could not represent the entire children and adolescent population. As our study was based on a dynamic open cohort of a natural population, selection bias still exists. It excluded more obese children and included children with lower BMI and weaker effects on HBP, while the effect of SO_2_ and CO on blood pressure was still observed.

## 5. Conclusions

In summary, our study supported a positive correlation between air pollution and HBP risks among children and adolescents, and a negative correlation between greenness and HBP risks. Meanwhile, the greenness reduces the risks of HBP attributed to SO_2_ and CO exposure among students, but its benefit was more effective for normal-weight participants than those obese. It was suggested that some targeted measures should be taken to reduce the specific gaseous pollutants. According to the characteristic of various districts, improving green space construction and preventing childhood obesity could reduce the burden of childhood HBP and subsequent cardiovascular disease risks.

## Data availability statement

The raw data supporting the conclusions of this article will be made available by the authors, without undue reservation.

## Ethics statement

The studies involving human participants were reviewed and approved by the Biomedical Ethics Committee of Peking University Health Science Center. Written informed consent to participate in this study was provided by the participants' legal guardian/next of kin.

## Author contributions

YZ and SC conceived the study design, performed the analysis, interpreted the findings, and wrote the manuscript. LC, YW, and JW prepared, analyzed, cleaned the data, interpreted the findings, and helped with manuscript preparation. TM, MC, and QM cleaned the data, interpreted the findings, and helped with manuscript preparation. JL, LW, WL, XL, JZ, and XW helped with manuscript preparation. JM and XG contributed to the conception of the work. YX and JZ worked on study design and data collection and helped with manuscript preparation. YD contributed to the conception of the work and helped with manuscript preparation. All authors read and approved the final manuscript.
