# Supplementary material for: Association of SO2/CO exposure and greenness with high blood pressure in children and adolescents: A longitudinal study in China
Source: Front Public Health. 2023 May 25;11:1097510. doi: 10.3389/fpubh.2023.1097510 (PMC10248062; doi:10.3389/fpubh.2023.1097510)
Supplement: Supplementary file 1 [file Table_1.DOCX]

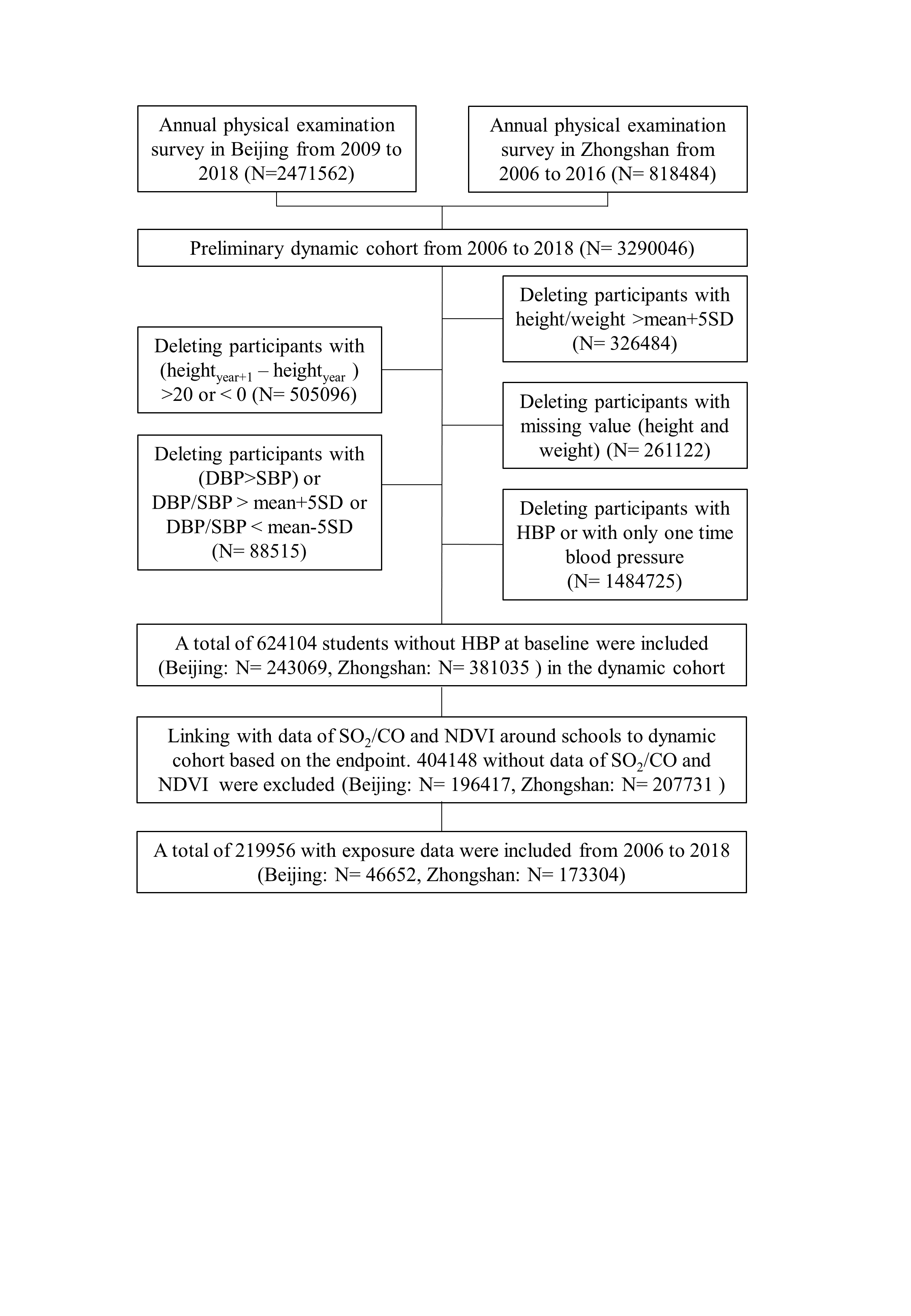


**Fig. A.1.** The flow chart.

| **Table A1**. Characteristics of included and excluded participants | | | |
| --- | --- | --- | --- |
| Characteristics | Included^†^  N=219956 | Excluded^†^  N=404148 | *P* value |
| Girls; n (%) | 96225(43.75) | 178573(44.19) | <0.001# |
| Boys; n (%) | 123731(56.25) | 225575(55.81) |  |
| Beijing; n (%) | 46652(21.21) | 196417(48.60) | <0.001# |
| Zhongshan; n (%) | 173304(78.79) | 207731(51.40) |  |
| Age (years); mean (SD) | 12.90(2.89) | 14.56(2.50) | <0.001^*^ |
| Years of follow-up; mean (SD) | 3.09(1.98) | 1.71(0.99) | <0.001^*^ |
| Height (cm); mean (SD) | 153.54(15.46) | 160.18(12.97) | <0.001^*^ |
| Weight (kg); mean (SD) | 45.65(15.68) | 52.37(14.91) | <0.001^*^ |
| BMI (kg/m2); mean (SD) | 18.80(3.82) | 20.05(3.85) | <0.001^*^ |

**Notes**: BMI = Body mass index. ^#^: Chi-Square test; ^*^: t-test. ^†^: There were 624104 children and adolescents with normal height, and weight, more than one record of blood pressure, and without HBP at the baseline, and 404148 among them without data of SO_2_/CO/NDVI were excluded, 219956 with complete exposure data were included.

**Table A2**: Intereaction analysis with GEE regression

| Outcome  variable | Interaction | Girls and Boys | | Girls | | Boys | |
| --- | --- | --- | --- | --- | --- | --- | --- |
|  |  | β (95%CI) | P value | β (95%CI) | P value | β (95%CI) | P value |
| SBP | SO_2_ × NDVI | -0.13 (-0.19, -0.06) | <0.001 | -0.05 (-0.14, 0.05) | 0.338 | -0.19 (-0.28, -0.1) | <0.001 |
|  | CO × NDVI | 0.37 (0.33, 0.41) | <0.001 | 0.41 (0.36, 0.46) | <0.001 | 0.38 (0.33, 0.43) | <0.001 |
| DBP | SO_2_ × NDVI | 0.05 (0.01, 0.09) | 0.013 | -0.01 (-0.06, 0.05) | 0.855 | 0.09 (0.04, 0.14) | <0.001 |
|  | CO × NDVI | 0.17 (0.15, 0.19) | <0.001 | 0.20 (0.17, 0.23) | <0.001 | 0.15 (0.12, 0.18) | <0.001 |

| **Table A3**: HR and AF of SO_2_/CO by NDVI | | | | | |
| --- | --- | --- | --- | --- | --- |
| Factor | High-level group of NDVI | |  | Low-level group of NDVI | |
|  | Boys | Girls |  | Boys | Girls |
| **SO_2_** |  |  |  |  |  |
| HR | 1.56(1.51,1.62) | 1.76(1.69,1.82) |  | 1.81(1.74,1.88) | 1.85(1.77,1.93) |
| AF(%) | 12.53(11.65,13.41) | 15.26(14.29,16.23) |  | 26.01(24.53,27.49) | 26.55(24.90,28.21) |
| **CO** |  |  |  |  |  |
| HR | 1.81(1.75,1.87) | 1.86(1.79,1.93) |  | 1.65(1.59,1.70) | 1.74(1.67,1.81) |
| AF(%) | 17.55(16.62,18.48) | 17.88(16.85,18.91) |  | 19.18(17.96,20.41) | 21.14(19.73,22.55) |

**Note**: HR, hazard ratio. HR of SO_2_ and CO concentration on HBP risk was calculated using Cox models, under the low-level and high-level green by gender, confounders adjusted. AF, attributable fraction. We calculated the AF of HBP risks attributed to SO_2_ and CO in the low-level and the high-level group of NDVI.


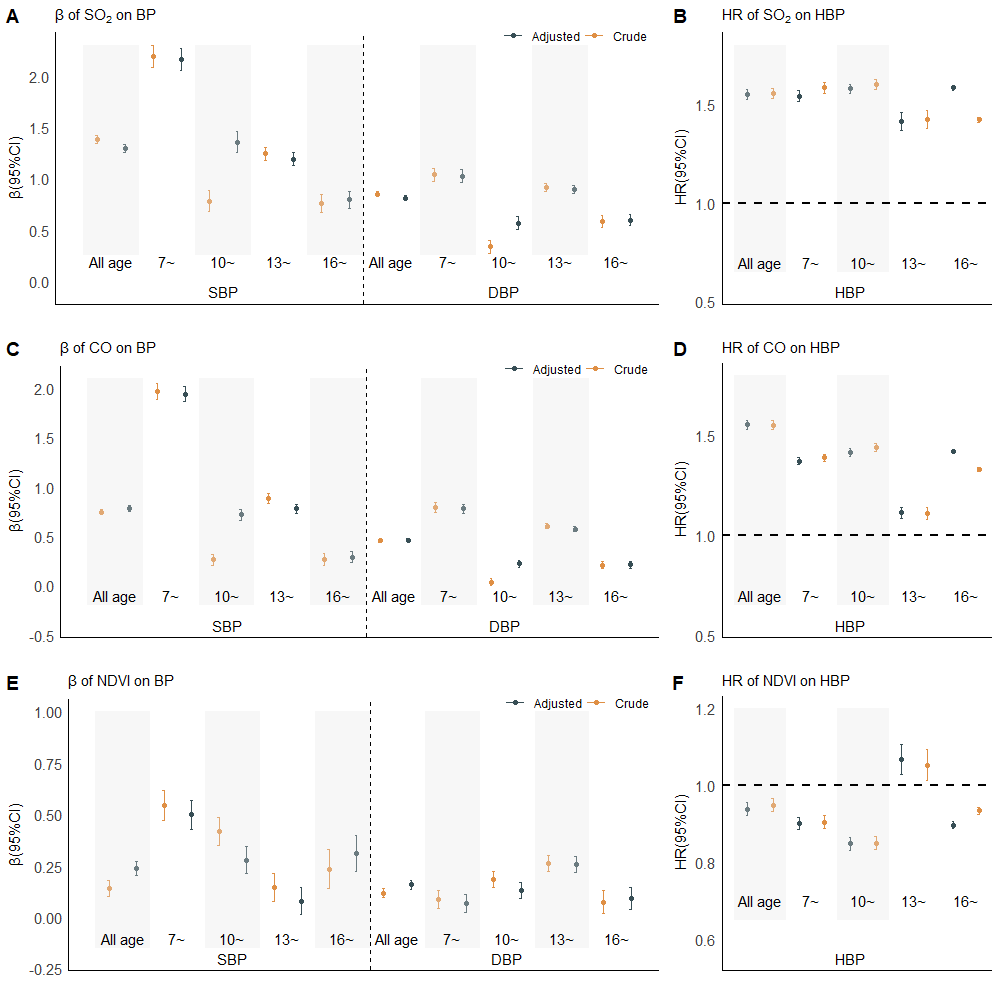


**Fig. A.2**. (A) Association between SO_2_ and BP by age. (B) Association between SO_2_ and HBP by age. (C) Association between CO and BP by age. (D) Association between CO and HBP by age. (E) Association between NDVI and BP by age. (E) Association between NDVI and HBP by age.


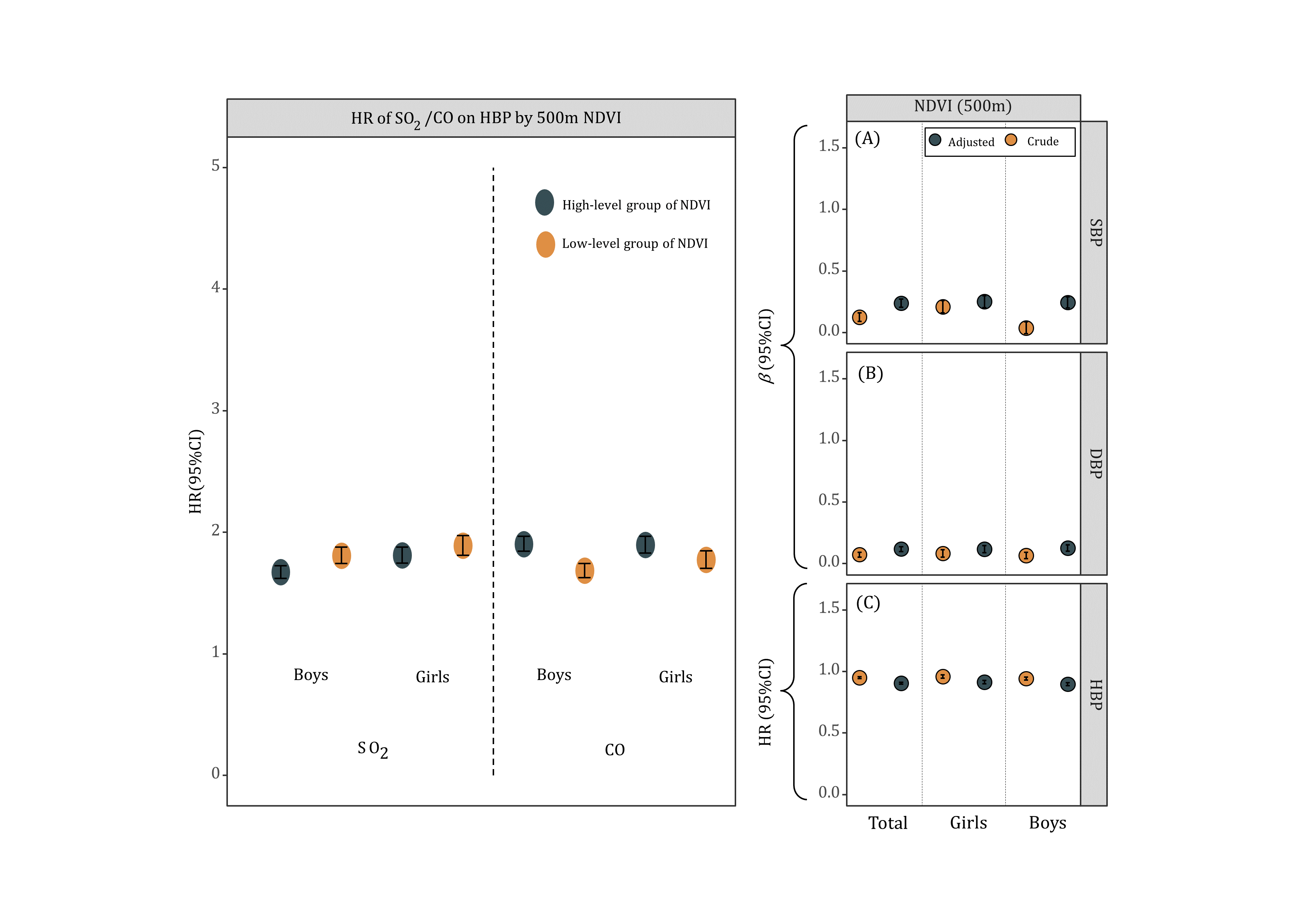


**Fig. A.3.** The left graph is the result of Cox proportional hazards models, where HBP was binary outcome variable, grouped by NDVI (500m radius). (A, B) Results of GEE models, NDVI (500m radius). (C) Results of Cox models, NDVI (500m radius).


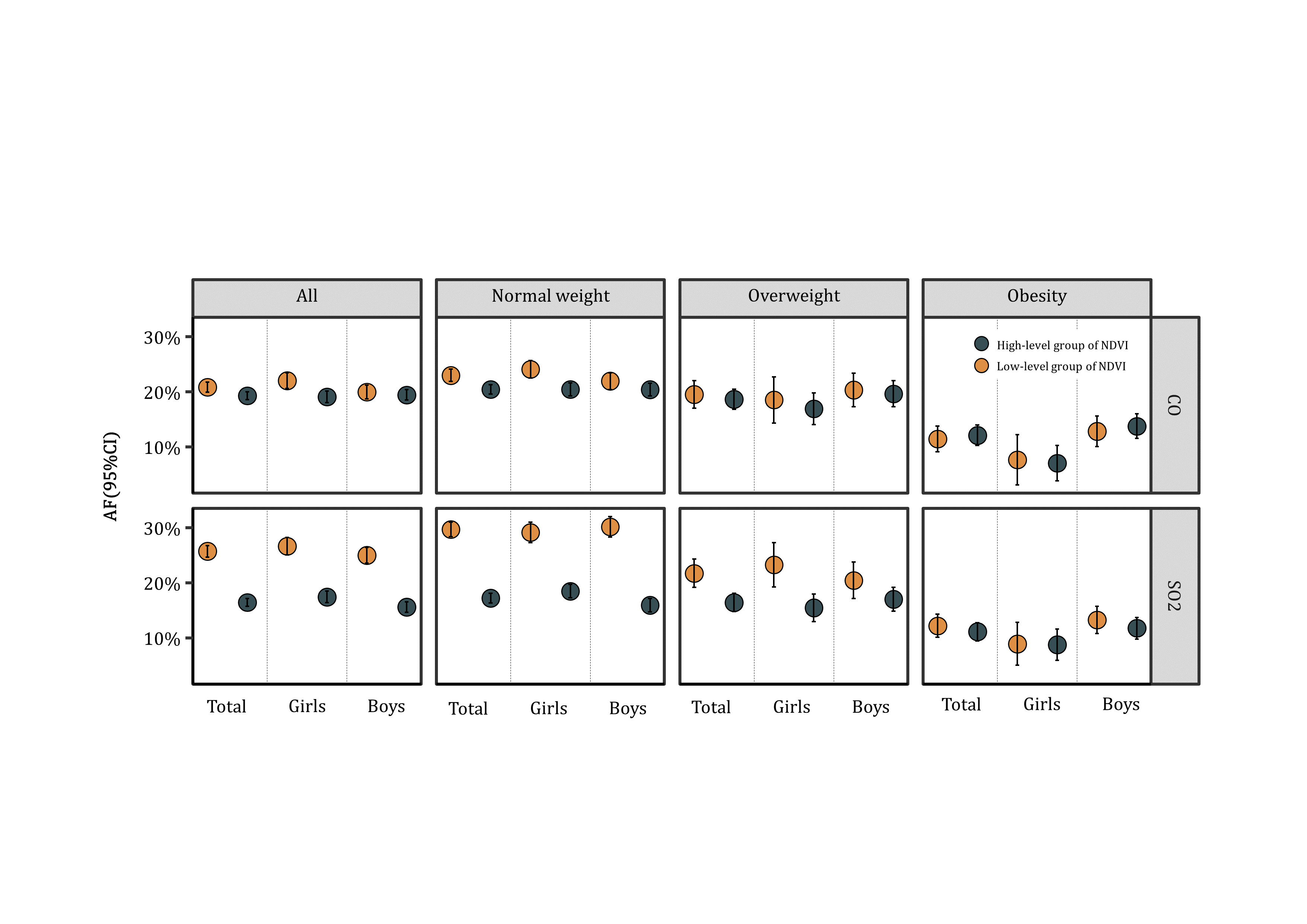


**Fig. A.4.** The attributable fraction of SO_2_ and CO on HBP in the low-level group and the high-level group of green by gender and BMI, NDVI (500m radius).


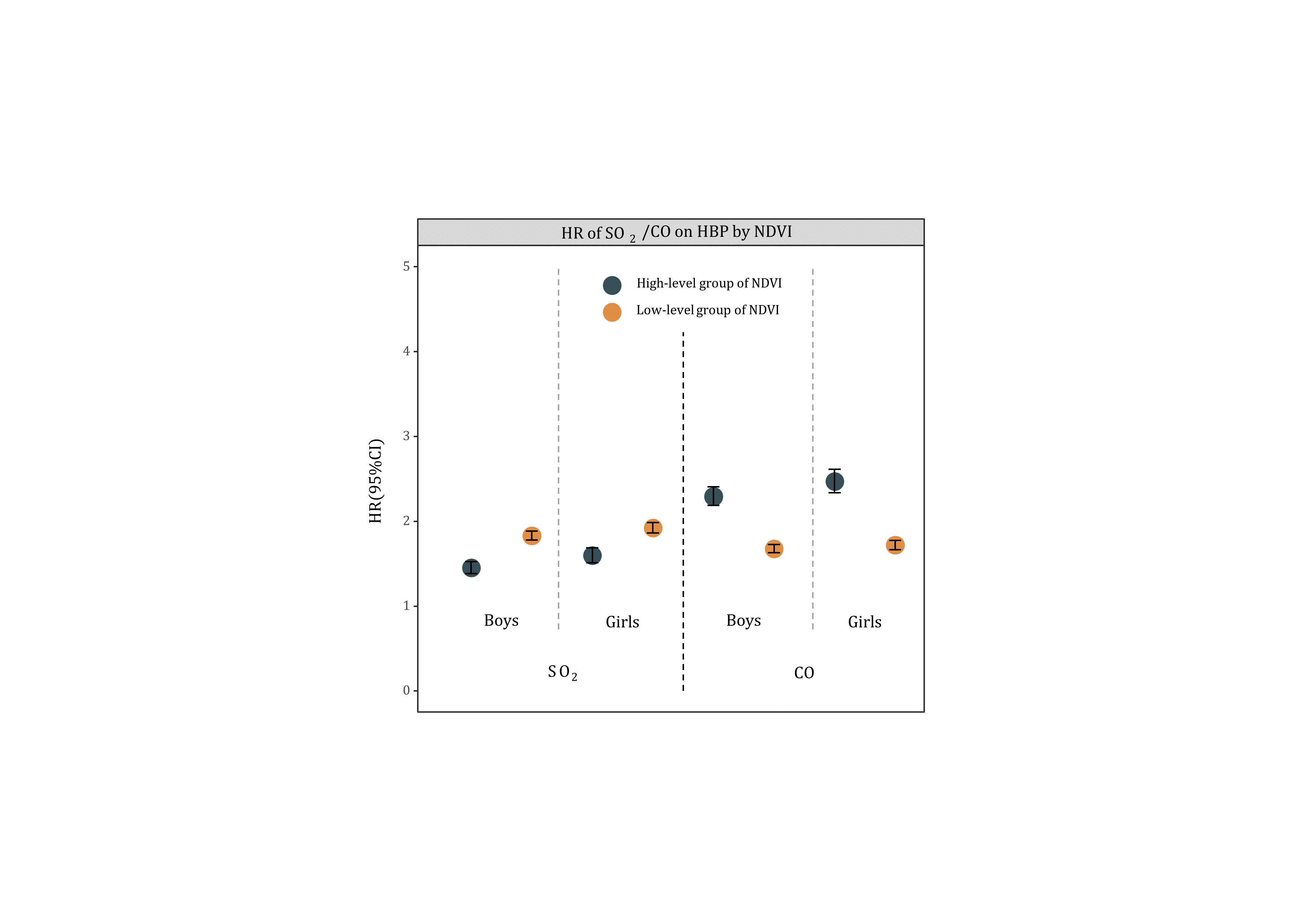


**Fig. A.5.** Result of Cox proportional hazards models, grouped by NDVI (1 km radius, cut-off = the third quartile).


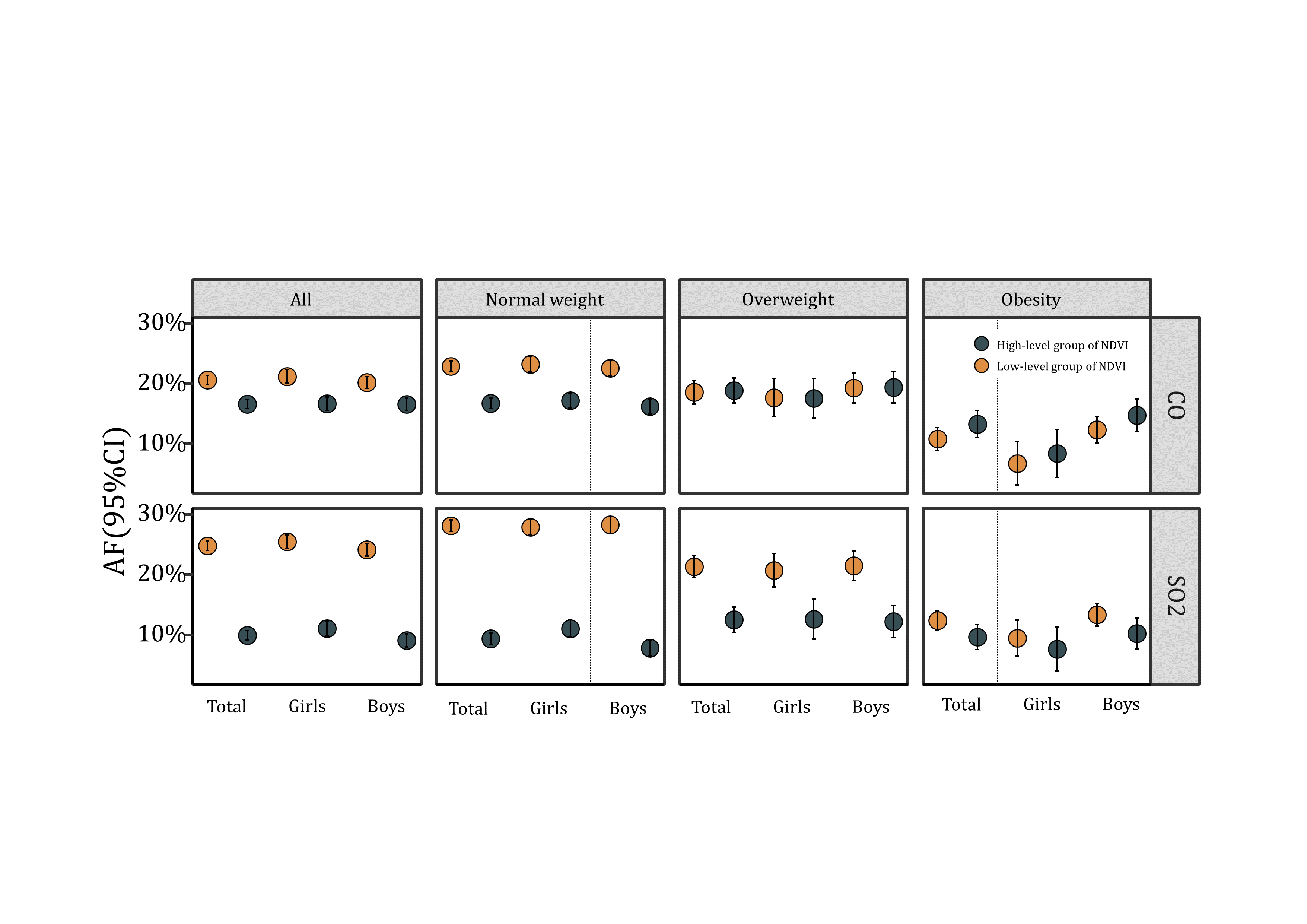


**Fig. A.6.** The attributable fraction of SO_2_ and CO on HBP in the low-level group and the high-level group of green by gender and BMI, NDVI (1 km radius, cut-off = the third quartile).


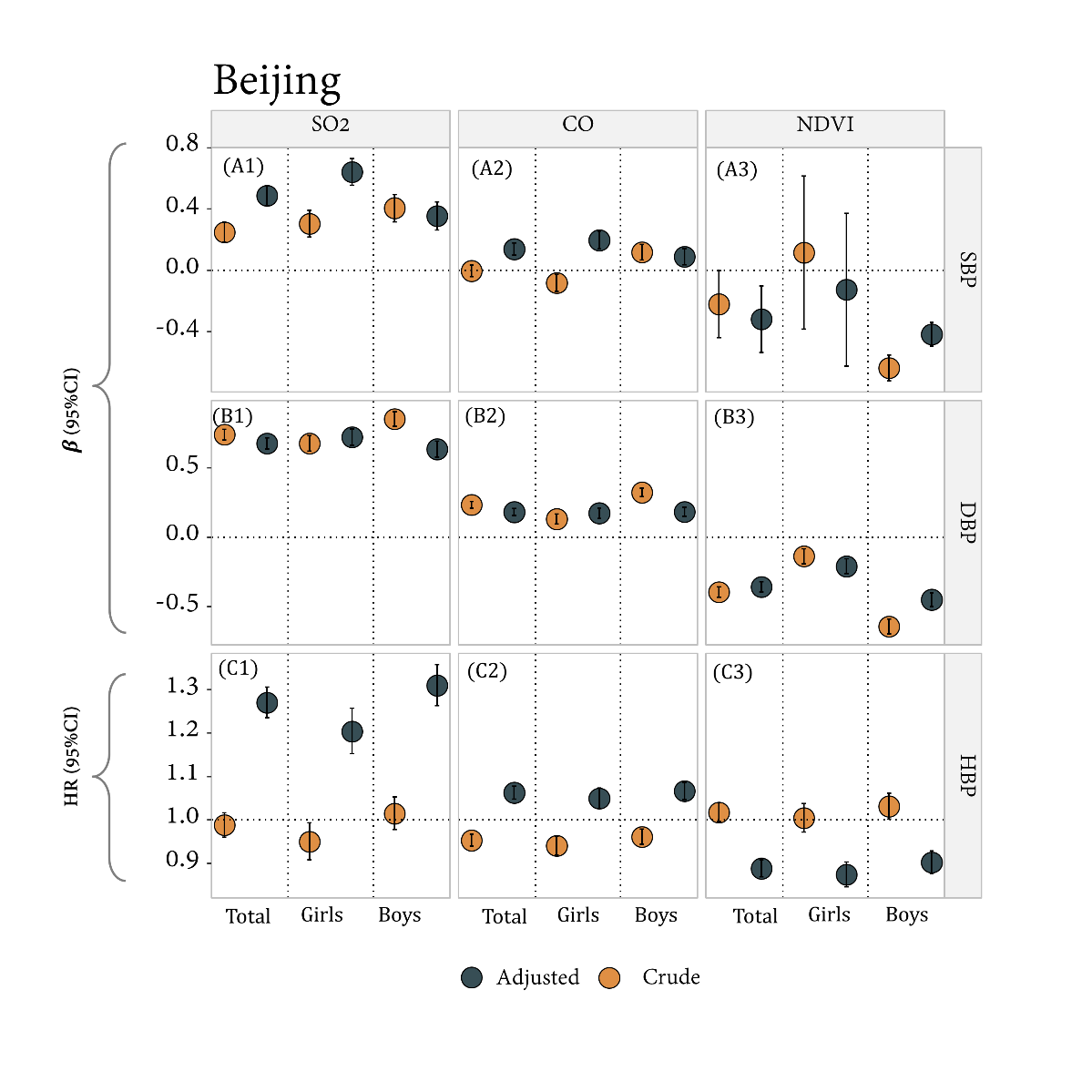


**Fig. A.7.** (A1~A3, B1~B3) The association between SO_2_/CO/NDVI and SBP/DBP (GEE model). (C1~C3) The quantitative association between SO_2_/CO/NDVI and HBP (Cox model). Estimates adjusted for age, BMI, and city and stratified for sex (total estimates also adjusted for sex). β=estimated coefficient. HR=hazard ratio.
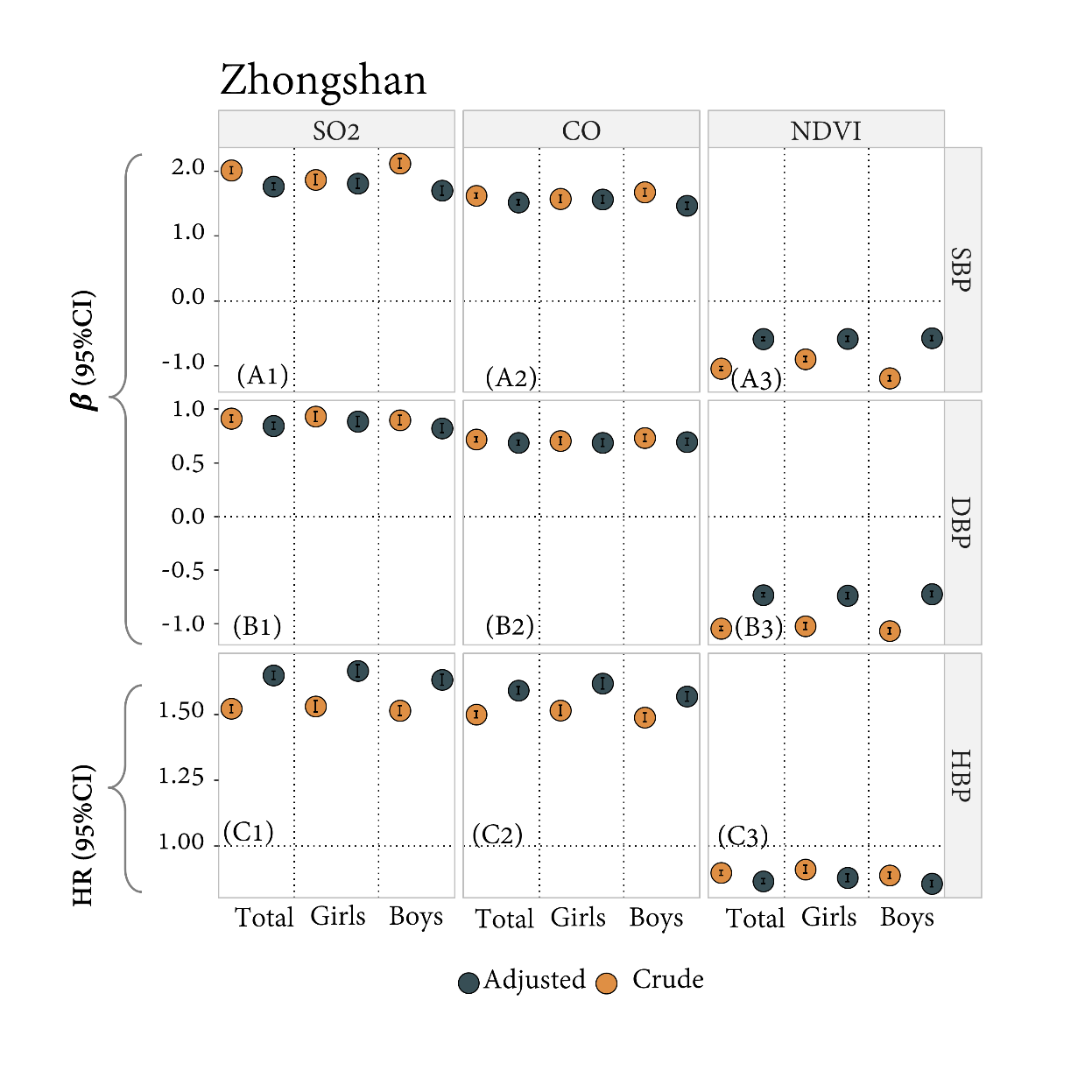


**Fig. A.8.** (A1~A3, B1~B3) The association between SO_2_/CO/NDVI and SBP/DBP (GEE model). (C1~C3) The quantitative association between SO_2_/CO/NDVI and HBP (Cox model). Estimates adjusted for age, BMI, and city and stratified for sex (total estimates also adjusted for sex). β=estimated coefficient. HR=hazard ratio.
